# Effects of Ketoconazole on the Pharmacokinetics of Ponatinib in Healthy Subjects

**DOI:** 10.1002/jcph.109

**Published:** 2013-06-25

**Authors:** Narayana I Narasimhan, David J Dorer, Katie Niland, Frank Haluska, Daryl Sonnichsen

**Affiliations:** 1 ARIAD Pharmaceuticals, Inc.Cambridge, MA, USA; 2 Sonnichsen Pharmaceutical AssociatesCollegeville, PA, USA

**Keywords:** BCR-ABL, ketoconazole, pharmacokinetics, ponatinib, tyrosine kinase inhibitor

## Abstract

Ponatinib is a BCR-ABL tyrosine kinase inhibitor (TKI) approved for the treatment of chronic myeloid leukemia and Philadelphia chromosome–positive acute lymphoblastic leukemia in patients resistant or intolerant to prior TKIs. In vitro studies suggested that metabolism of ponatinib is partially mediated by CYP3A4. The effects of CYP3A4 inhibition on the pharmacokinetics of ponatinib and its CYP3A4-mediated metabolite, AP24567, were evaluated in a single-center, randomized, two-period, two-sequence crossover study in healthy volunteers. Subjects (N = 22) received two single doses (orally) of ponatinib 15 mg, once given alone and once coadministered with daily (5 days) ketoconazole 400 mg, a CYP3A4 inhibitor. Ponatinib plus ketoconazole increased ponatinib maximum plasma concentration (C_max_) and area under the concentration–time curve (AUC) compared with ponatinib alone. The estimated mean ratios for AUC_0–∞_, AUC_0–t_, and C_max_ indicated increased exposures to ponatinib of 78%, 70%, and 47%, respectively; exposure to AP24567 decreased by 71%. Exposure to AP24567 was marginal after ponatinib alone (no more than 4% of the exposure to ponatinib). These results suggest that caution should be exercised with the concurrent use of ponatinib and strong CYP3A4 inhibitors and that a ponatinib dose decrease to 30 mg daily, from the 45 mg daily starting dose, could be considered.

The use of tyrosine kinase inhibitors (TKIs) that target BCR-ABL is a well-established and highly effective strategy for sustained disease control in chronic myeloid leukemia (CML). Mutations in the kinase domain of BCR-ABL that impede effective inhibitor binding are the primary mechanism of resistance to currently available agents;^1^ in particular, the T315I gatekeeper mutant is resistant to all approved BCR-ABL inhibitors.^2^–^4^

Ponatinib (AP24534) is a novel pan–BCR-ABL inhibitor developed using a computational and structure-based approach.^5^–^7^ It was designed to bind to and inhibit native BCR-ABL and BCR-ABL mutants that can arise during treatment with other TKIs and cause resistance, including the T315I mutant.^6^ Based on results of phase 1 and phase 2 clinical trials in patients with CML and Philadelphia chromosome–positive acute lymphoblastic leukemia (Ph^+^ ALL),^8^,^9^ ponatinib (45 mg once daily) has been approved in the United States for the treatment of patients with CML and Ph^+^ ALL that is resistant or intolerant to prior TKI therapy.^10^

Preclinical studies have evaluated the oral bioavailability and metabolic profile of ponatinib. Absolute oral bioavailability is 54% in rats and 20% in monkeys. Studies conducted using human liver microsomes found that ponatinib is a substrate of cytochrome P450 (CYP)3A4/5 and, to a lesser extent, CYP2C8 and CYP2D6. On the basis of these studies, the major metabolic pathways of ponatinib were identified as *N*-demethylation (to form AP24567, a 4-fold less-potent metabolite) and *N*-oxidation (to form AP24734) mediated by CYP3A4/5. However, in vivo radiolabeled studies later found that a pharmacologically inactive metabolite (AP24600), formed through esterase/amidase-mediated hydrolysis of the amide bond in ponatinib, is the major circulating metabolite in humans.

In patients administered ponatinib orally once-daily, ponatinib was readily absorbed, with maximum plasma levels observed approximately 4 hours postdose.^8^ Following initial dose and under steady-state conditions, various measures of ponatinib exposure (maximum plasma concentration \C_max_] and area under the concentration–time curve \AUC]) were approximately dose-proportional (doses ranged from 2 to 60 mg). The elimination half-life of ponatinib 45 mg was approximately 22 hours. After a single 45-mg oral dose of 14C-labeled ponatinib was administered to healthy volunteers, 5% of total radioactivity was recovered in urine and 87% in feces; thus, fecal elimination is the major excretory pathway of ponatinib.^10^ With once-daily ponatinib 45 mg, an approximately 1.5-fold accumulation of AUC from first dose to steady-state was observed.^8^ Daily doses of 30 mg achieved steady-state trough plasma concentrations that surpassed 40 nM, a concentration sufficient to inhibit viability of cells expressing all BCR-ABL mutants tested—including the T315I gatekeeper mutant—and to suppress the emergence of mutant clones in preclinical mutagenesis assays.^6^,^8^ Once-daily ponatinib 45 mg achieved geometric mean (GeoMean) steady-state trough and C_max_ concentrations of 34.2 ng/mL (64 nM) and 77.4 ng/mL (145 nM), respectively, substantially above the 40 nM target.^8^

CYP3A4 is the major P450 isoenzyme involved in the microsomal biotransformation of imatinib,^11^ nilotinib,^12^ and dasatinib.^13^ Ketoconazole selectively inhibits CYP3A4 in vitro and in vivo.^14^ Ketoconazole is the model drug that is used for evaluation of CYP3A4 inhibition on the pharmacokinetics (PK) of oral drugs metabolized by CYP3A4.^15^ Because CYP3A4 is also involved in the metabolism of ponatinib, this study was conducted to investigate the PK of ponatinib in combination with ketoconazole as a model for drug interactions involving CYP3A4.^15^

## Methods

### Subjects

Healthy subjects were enrolled in this study. Before participation, male patients or postmenopausal or surgically sterile female patients aged 18–55 years (inclusive) underwent medical screening that included medical history, physical examination, vital signs, electrocardiogram (ECG) results, and routine laboratory tests. Subjects were required to have a body mass index between 18.0 and 33.0 kg/m^2^ and a minimum weight of 50.0 kg at screening. Subjects with clinically significant illness; history of use of any investigational or prescription drug within 30 days before first study drug administration or over-the-counter drugs within 72 hours before first study drug administration; a history of drug or alcohol abuse; abnormalities in vital signs or laboratory values; or clinically significant abnormalities on physical examination, medical history, or 12-lead ECG (i.e., QTcB >440 ms for male subjects and >450 ms for female subjects) were excluded from the study. Subjects were asked to abstain from foods known to influence CYP metabolism, including grapefruit or grapefruit-containing products, pomegranate, pomelo, and star fruit juice/products, as well as foods containing poppy seeds, Seville oranges, and/or drinks and foods containing quinine from 72 hours before first drug administration until the end of the study.

### Study Design

This study was performed at Kendle Early Stage (Toronto, Canada), an independent contract research organization, and was sponsored by ARIAD Pharmaceuticals, Inc. (Cambridge, MA, USA). This was a single-center, open-label, randomized, two-period, two-sequence crossover, inpatient/outpatient study consisting of two treatment periods separated by a minimum 14-day washout period between ponatinib doses. Each subject participated in the study for 6–8 weeks, including a screening evaluation within 3 weeks before first ponatinib administration, two 4-night/5-day inpatient treatment periods, one outpatient visit per treatment period, and a safety follow-up visit.

Each subject received two single doses of ponatinib 15 mg, once given alone and once coadministered with once daily doses of ketoconazole 400 mg, for 5 days. Subjects were randomly allocated to a treatment sequence on day −1 of the first treatment period. During the ponatinib alone schedule, subjects were admitted on day −1, received a single 15-mg ponatinib dose with approximately 240 mL of water in the morning on day 1, and were discharged on day 4 after 72-hour assessments were complete. Subjects returned to the research site for an outpatient visit for 96-hour sample collection and safety assessments. For the ponatinib with ketoconazole schedule, subjects were admitted to the research site on day −1 and received the first ketoconazole dose (400 mg as two 200-mg tablets) in the evening on day −1, approximately 12 hours before the scheduled dose of ponatinib. In the morning on day 1, subjects received a single 15-mg ponatinib dose in combination with the second dose of ketoconazole 400 mg. Subjects continued to receive once daily 400 mg ketoconazole doses in the morning on days 2–4 and were discharged on day 4 after completion of ketoconazole dosing and 72-hour assessments. Subjects returned for an outpatient visit for 96-hour sample collection and safety assessments. All subjects returned for a safety follow-up visit within 10–14 days of last ponatinib administration.

For both regimens, subjects were required to fast for 10 hours prior to ponatinib dosing and for 4 hours post dosing on day 1. During the ponatinib + ketoconazole regimen, subjects were required to fast for 1 hour before and after ketoconazole dosing on day −1 and days 2–4. The dose of ponatinib (15 mg) was selected for coadministration with ketoconazole to allow for an appropriate safety margin between potential plasma exposures resulting from CYP3A4 inhibition and those supported by previous experience in patients with cancer. The ketoconazole dose was selected in accordance with literature recommending a 400 mg once-daily regimen as necessary to obtain maximum inhibition of CYP3A within 2 days of dosing^16^ and has resulted in significant inhibition of clearance for another CYP3A4 substrate.^17^

The primary end point of the study was assessment of the PK parameters of ponatinib; the secondary end point of the study was the evaluation of the PK parameters of AP24567, a CYP3A4-mediated metabolite of ponatinib. Levels of AP24734, another CYP3A4-mediated metabolite, were approximately 3-fold lower than levels of AP24567; as a result, the PK parameters of AP24734 were not evaluated. Adverse events, vital signs, clinical laboratory assessments, physical exam results, and 12-lead ECG results were also collected. All patients provided signed informed consent and the study was conducted in accordance with the Guidelines for Good Clinical Practice and the Declaration of Helsinki. The protocol was approved by Institutional Review Board Services Ontario.

### Blood Sampling

Blood samples for determination of plasma ponatinib and AP24567 were obtained through 96 hours after dosing. At the time of each ponatinib and AP24567 determination in plasma approximately 4 mL of blood was collected into prechilled K2-EDTA tubes (or the equivalent), labeled, processed, and stored at −20 ± 5°C. The time points analyzed were predose and 0.5, 1, 2, 4, 5, 6, 8, 12, 24, 36, 48, 72, and 96 hours after dosing.

### Plasma Ponatinib and AP24567 Analyses

Concentrations of ponatinib and AP24567 were determined in plasma by liquid chromatography/tandem mass spectrometry (LC/MS/MS). Calibration standards (nine samples, covering the range 0.5–250 ng/mL for ponatinib and 0.1–50 ng/mL for AP24567) and quality control (QC) standards (four samples, covering the range 0.5–200 ng/mL for ponatinb and 0.1–40 ng/mL for AP24567) were prepared by spiking ponatinib and AP24567 solutions of known concentrations into human plasma; the lower limit of quantitation (LOQ) for ponatinib was 0.5 ng/mL and the LOQ for AP24567 was 0.1 ng/mL. Aliquots (0.15 mL) of study plasma samples, calibration standards, QC standards, and control samples were transferred to individual wells of a 96-well block. A 0.05 mL solution containing deuterated internal standards (D_3_-ponatinb and D_4_-AP24567) was added to the plasma samples, followed by addition of 0.4 mL of 10 mM ammonium acetate buffer, pH 7.0. The samples were vortexed and centrifuged. The supernatants (0.6 mL) were passed through a preconditioned solid phase extraction tube (Isolute, 100 mg, Biotage). The tubes were washed with methanol and the analytes were eluted into another 96-well block using 0.4 mL of 98/2-methanol/formic acid solution. The eluates were evaporated to dryness, reconstituted with 0.1 mL of a reconstitution solution (50:50 methanol/1000:10 water/1 M ammonium bicarbonate, pH 10). The final extracts were analyzed by a validated LC/MS/MS method. Data were collected in positive ion mode. Calculated correlations are based on peak area ratios of the analyte to its respective internal standard. The smoothing width was three. The peak area for each analyte transition was divided by the peak area for the corresponding IS transition. The calibration curves for ponatinib and AP24567 were fit using a linear regression, weighted 1/x^2^. Intra- and inter-assay precision (% coefficient of variation \CV]) was ≤9.4% for ponatinib and ≤10.3% for AP24567; the intra- and inter-assay accuracy (% relative error \RE]) was −10.4% to 10.5% for ponatinib and −13.8% to 6.3% for AP24567.

### Pharmacokinetic Analysis

Pharmacokinetic parameters were determined using noncompartmental methods with WinNonlin Version 5.2 (Pharsight Corporation, Mountain View, CA) for ponatinib and the metabolite AP24567. The following parameters were determined: maximum plasma concentration (C_max_); time to maximal plasma concentration (t_max_); area under the plasma concentration versus time curve from time 0 to the time of last quantifiable concentration (AUC_0–t_); area under the plasma concentration versus time curve from time 0 to time infinity (AUC_0–∞_); elimination half-life (t_1/2_); apparent or fractional plasma clearance (CL/F; for ponatinib only); and apparent distribution volume in the terminal elimination phase (V/F; for ponatinib only).

### Statistical Analysis

Based on an estimated intrasubject variability in ponatinib PK of 25%, derived from phase 1 PK data in cancer patients,^8^ and with a sample size of 20 subjects, the power for concluding equivalence in ponatinib C_max_ and AUC within the limits of 0.80 and 1.25, using a 90% confidence interval (CI), was expected to exceed 80% at the 0.05 significance level, assuming a 5% or less difference between treatments (ponatinib + ketoconazole vs. ponatinib alone). To ensure that at least 20 subjects successfully completed the study, 24 subjects were to be enrolled.

Plasma concentration–time profiles following ponatinib alone and when ponatinib and ketoconazole were coadministered were generated; the derived PK parameters were listed and tabulated. Inferential tests were conducted to evaluate the effect of ketoconazole coadministration on the bioavailability of ponatinib and AP24567. Mixed-model analyses of variance (ANOVA) were performed on the natural log transformed (Ln-transformed) PK parameters AUC_0–∞_, AUC_0–t_, and C_max_. The ANOVA model for a two-period crossover design included sequence, treatment (with or without ketoconazole coadministration), and period as fixed effects and subject nested within sequence as a random effect. Sequence was tested using subject nested within sequence as the error term, at a 10% level of significance; all other main effects were tested using the residual error (error mean square). Each ANOVA included calculation of least square mean (LSM), the difference between treatment LSM, and the standard error associated with the difference. These were calculated using the SAS® Mixed procedure. Ratios of LSM were calculated using the exponential of the difference between treatment LSM from the analyses on the Ln-transformed AUC_0–t_, AUC_0–∞_, and C_max_. An absence of a drug interaction was indicated if the 90% CIs for the ratio of population geometric means between ketoconazole + ponatinib and ponatinib alone was contained in the equivalence limits of 80% to 125%. For each ANOVA, only paired data sets were used (i.e., for each PK parameter, the subjects’ data comprised a reliable value for both treatments). Differences between ponatinib t_max_, as well as those between AP24567 t_max_ obtained after ponatinib administration with and without ketoconazole coadministration, were evaluated by nonparametric testing of nontransformed data (Wilcoxon signed-rank test).

## Results

### Subjects

A total of 24 subjects were randomized to the treatment phase; one withdrew consent prior to dosing, yielding 23 subjects who took at least one dose of the study drug (safety population). One subject withdrew consent on day 3 of the second treatment period, yielding 22 who completed the study (PK population; Table 1). All subjects included in the PK set were male and more than half (68.2%) were white. The median age of subjects was 42 (range: 22–53) years and the mean body mass index was 25.36 kg/m^2^. No prior or concomitant medication use was reported for any subject.

**Table 1:** Baseline Characteristics

Demographic characteristic	PK population (N = 22)
Age, y	
Median (range)	42 (22–53)
Gender, n (%)	
Male	22 (100.0)
Race, n (%)	
White	15 (68.2)
Black/African American	5 (22.7)
Asian	2 (9.1)
Weight (kg)	
Mean (SD), (range)	77.46 (12.5), (59.3–110.4)
BMI, (kg/m^2^)	
Mean (SD), (range)	25.36 (3.0), (18.6–29.3)

BMI, body mass index; PK, pharmacokinetic; SD, standard deviation.

### Ponatinib and AP24567 Pharmacokinetics

**Figure 1:**
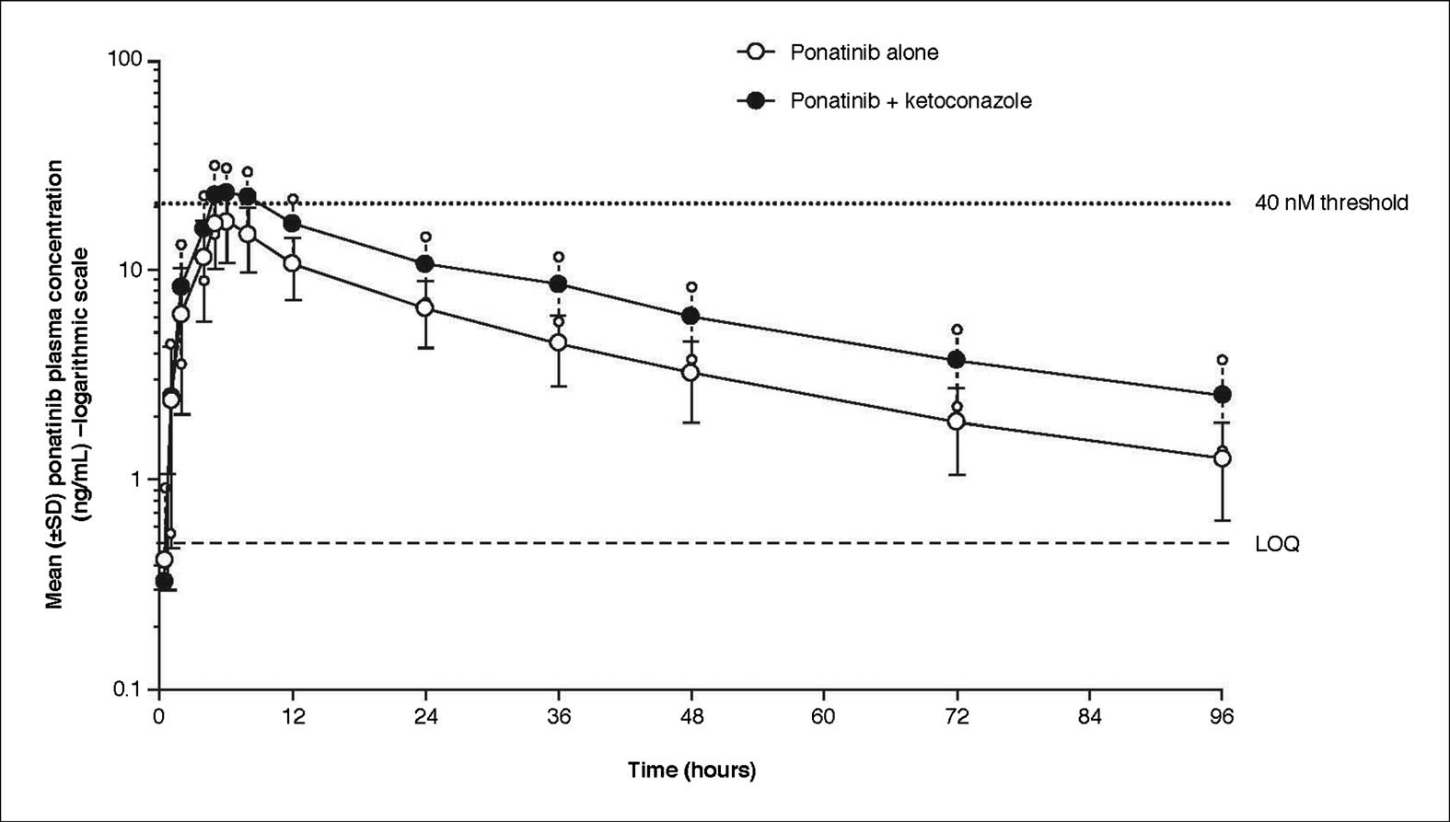
Mean (±SD) ponatinib plasma concentrations (logarithmic scale) versus time for ponatinib alone and ponatinib + ketoconazole. Ponatinib was administered as a single oral 15-mg dose, once given alone and once coadministered with daily doses of ketoconazole 400 mg, for 5 days. The dashed line depicts the lower limit of quantitation (LOQ) for ponatinib (0.5 ng/mL), and the dotted line represents the 40 nM threshold concentration sufficient to inhibit all BCR-ABL mutants tested and to suppress the emergence of mutant clones in preclinical mutagenesis assays.^8^ Error bars represent the standard deviation (SD).

**Figure 2:**
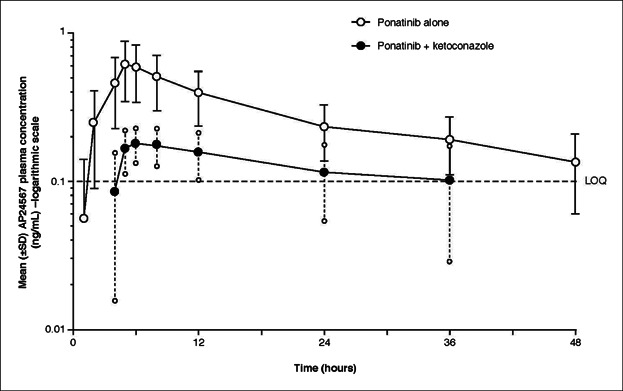
Mean (±SD) AP24567 plasma concentrations (logarithmic scale) versus time for ponatinib alone and ponatinib + ketoconazole. Ponatinib was administered as a single oral 15-mg dose, once given alone and once coadministered with daily doses of ketoconazole 400 mg, for 5 days. The dashed line depicts the lower limit of quantitation (LOQ) for AP24567 (0.1 ng/mL). Error bars represent the standard deviation (SD).

The mean concentration–time profiles of ponatinib and AP24567 when administered alone or in combination with ketoconazole are shown in Figures 1 and 2. Independent of ketoconazole coadministration, the mean plasma concentrations of ponatinib following a single oral dose of 15 mg reached a maximum at approximately 5–6 hours after oral administration; however, plasma concentrations of ponatinib were higher during ketoconazole coadministration. Ponatinib was quantifiable in all subjects at 96 hours after dosing in the presence and absence of coadministered ketoconazole.

For ponatinib, mean and median t_max_ were similar in the presence or absence of coadministered ketoconazole (Table 2). Maximum concentration was observed at approximately 6 hours post dose (range: 2–8 hours for ponatinib alone and 5–8 hours for ponatinib + ketoconazole). The observed GeoMean C_max_ was 16.08 ng/mL after ponatinib alone and 23.59 ng/mL after the ponatinib + ketoconazole regimen, an increase of 47%. The AUC_0–t_ and AUC_0–∞_ values increased from 413.5 and 472.2 h × ng/mL to 700.7 and 785.3 h × ng/mL, respectively, when ponatinib was coadministered with ketoconazole. Ponatinib plasma concentrations declined in a multiexponential fashion; ponatinib terminal elimination was not essentially affected by coadministration with ketoconazole.

**Table 2:** Ponatinib and AP24567 Pharmacokinetic Parameters

	t_max_, h^a^	C_max_, ng/mL (%CV, n)^b^	AUC_0–t_, h × ng/mL (%CV, n)^b^	AUC_0–∞_, h × ng/mL (%CV, n)^b^	t_1/2_, h^c^	CL/F, L/h (%CV, n)^b^	V/F, L (%CV, n)^b^
Ponatinib							
Ponatinib alone (N = 22)	6.0 \2.0–8.0], (23.5, 22)	16.1 (36.0, 22)	413.5 (35.8, 22)	472.2 (38.0, 22)	35.3 (12.4, 22)	31.8 (45.6, 22)	1604 (51.0, 22)
Ponatinib + ketoconazole (N=22)	6.0 \5.0–8.0], (20.4, 22)	23.6 (30.7, 22)	700.7 (34.4, 22)	785.3 (35.0, 19)	37.4 (14.7, 21)	19.1 (33.9, 19)	1001 (33.5, 19)
AP24567							
Ponatinib alone (N = 22)	5.0 \4.0–12.0], (28.1, 22)	0.568 (40.8, 22)	12.3 (55.6, 22)	23.9 (50.8, 22)	37.6 (39.7, 13)	ND	ND
Ponatinib + ketoconazole (N = 22)	6.0 \5.0–12.0], (30.9, 22)	0.185 (27.1, 22)	3.5 (76.0, 22)	ND	ND	ND	ND

AUC_0–∞_, area under the plasma concentration versus time curve from time 0 to infinity; AUC_0–t_, area under the plasma concentration versus time curve from time 0 to the time t of last quantifiable concentration; CL/F, apparent fractional plasma clearance; C_max_, maximal observed plasma concentration; CV, coefficient of variation; N, number of subjects receiving treatment; n, number of subjects with contributing data; ND, not determined; t_1/2_, terminal elimination half-life; t_max_, time to maximal plasma concentration; V/F, apparent distribution volume in the terminal elimination phase.

a Value for t_max_ is presented as median \range], (%CV, n).

b Geometric mean, %CV, and number of subjects with contributing data are shown.

c Mean, %CV, and number of subjects with contributing data are shown.

The median t_max_ for AP24567 increased from 5 to 6 hours when ponatinib was coadministered with ketoconazole. The GeoMean C_max_ was 0.5684 ng/mL after ponatinib alone (the reference) and 0.1850 ng/mL after the ponatinib + ketoconazole regimen. A decrease was also observed for AUC_0–t_ values when ketoconazole was coadministered with ponatinib. The AUC_0–∞_ could not be derived in most instances, as the elimination rate could not be reliably estimated. Exposure to the metabolite was marginal compared with ponatinib for both treatments (maximum 4% when comparing observed GeoMean C_max_ and AUC_0–t_ values for the parent with those for the metabolite for the ponatinib alone regimen).

Statistical analysis demonstrated a significant effect of ketoconazole coadministration on the relative bioavailability of ponatinib and AP24567 (Table 3). Estimated mean ratios of AUC_0–∞_, AUC_0–t_, and C_max_ indicated increased exposures to ponatinib of 78%, 70%, and 47%, respectively, whereas exposure to AP24567 decreased by 71%. Wilcoxon signed-rank test outcomes for t_max_ indicated that ponatinib t_max_ was not affected by concomitant use of ketoconazole (*P* = 0.1143), while AP24567 t_max_ was delayed 1 hour with ketoconazole coadministration (*P* = 0.0041).

### Safety

Overall, ponatinib 15 mg was generally well tolerated both alone and in combination with ketoconazole. Eleven of 23 subjects experienced at least one treatment-emergent adverse event (TEAE); all TEAEs were mild and resolved without intervention. The overall incidence of TEAEs was higher with ponatinib administered alone (n = 8) than with ponatinib coadministered with ketoconazole (n = 5). Nine subjects experienced at least one drug-related AE; the incidence of drug-related AEs was similar between ponatinib administered alone (n = 6) and ponatinib coadministered with ketoconazole (n = 5). No deaths or other severe AEs were reported and no subjects discontinued due to a TEAE. There were no serious AEs.

The most common TEAEs with ponatinib alone were somnolence and increased lipase (2 \8.7%] of 23 subjects each), while somnolence and headache (2 \8.7%] of 23 subjects each) were the most common TEAEs observed following administration of ponatinib with ketoconazole. Other TEAEs occurred only in individual subjects. Mean laboratory parameters were within normal ranges at all visits and few treatment-related effects were observed. No treatment-related or clinically significant vital sign, ECG, or physical examination findings were reported during the study.

**Table 3:** ANOVA Results for Ponatinib and AP24567 Test to Reference Outcomes: PK Set

Reference treatment	Test treatment	Ln-transformed parameter	Estimated mean ratio (T/R), %	90% CI					
				Lower limit	Upper limit
Ponatinib					
Ponatinib alone	Ponatinib + ketoconazole	AUC_0–∞_	178.02	166.24	190.63
		AUC_0–t_	170.07	159.45	181.39
		C_max_	146.57	132.80	161.76
AP24567					
Ponatinib alone	Ponatinib + ketoconazole	AUC_0–t_	29.16	20.02	42.48
		C_max_	32.17	27.77	37.25

AUC_0-∞_, area under the plasma concentration versus time curve from time 0 to infinity; AUC_0-t_, area under the plasma concentration versus time curve from time 0 to the time t of last quantifiable concentration; CI, confidence interval; C_max_, maximal observed plasma concentration; Ln-transformed, natural log transformed; T/R, test/reference.

## Discussion

Preclinical data suggested that the isoenzyme CYP3A4 is implicated in the human metabolism of ponatinib. In this study, the potent inhibitor of CYP3A4, ketoconazole, was used to examine the contribution of this isoenzyme to ponatinib clearance in humans.

In 22 evaluable healthy subjects, coadministration of single-dose ponatinib 15 mg with multiple doses of ketoconazole 400 mg increased both ponatinib C_max_ and AUC compared with ponatinib administered alone. The estimated mean ratios for AUC_0–∞_, AUC_0–t_, and C_max_ indicated increased exposures to ponatinib of 78%, 70%, and 47%, respectively, while exposure to the CYP3A-mediated metabolite (AP24567) decreased by approximately 70%. Assuming dose-proportionality and a 78% average increase in ponatinib exposure, coadministration of a 45 mg daily dose of ponatinib with a strong CYP3A4 inhibitor would result in ponatinib exposure similar to that of an 80 mg daily dose (45 mg × 178% = 80.1 mg). Ponatinib 60 mg daily exceeded the maximum tolerated dose in a phase 1 study; therefore, a dose reduction to 30 mg daily (30 mg × 178% = 53.4 mg), with close monitoring for signs of possible increased toxicity, is recommended when ponatinib is coadministered with a strong CYP3A4 inhibitor.

As noted, initial in vitro studies conducted using human liver microsomes and hepatocytes identified the major metabolic pathways of ponatinib to be CYP3A4/5-mediated *N*-demethylation (to form AP24567) and *N*-oxidation (to form AP24734). However, later in vivo studies found that AP24600, a pharmacologically inactive metabolite formed through esterase/amidase-mediated hydrolysis of the amide bond in ponatinib after oral administration, is the major circulating metabolite in humans. Overall, this study demonstrated a statistically significant, albeit moderate (<2-fold), effect of ketoconazole coadministration on the relative bioavailability of ponatinib, consistent with CYP3A4 as a secondary pathway for ponatinib metabolism.

The effects of ketoconazole-mediated CYP3A4 inhibition on the metabolism of ponatinib compare favorably with those of other approved TKIs for the treatment of CML. Following coadministration of imatinib with a single dose of ketoconazole 400 mg, imatinib C_max_ increased by 1.3-fold; whereas, AUC_0–24_ and AUC_0–∞_ increased by 1.4-fold and apparent clearance decreased by 1.4-fold. There was no clear impact of single-dose ketoconazole on the t_1/2_ of imatinib; however, the impact of multiple-dose ketoconazole on imatinib exposure is unknown.^18^ Coadministration of dasatinib with multiple-dose ketoconazole (200 mg twice daily) resulted in an increase in C_max_ and AUC by 3.6- and 4.8-fold, respectively; t_1/2_ was prolonged by 5.4 hours.^19^ Similarly, CYP3A4 inhibition of nilotinib with multiple-dose ketoconazole (400 mg once daily) resulted in increases of 1.8- and 3-fold for AUC_0–t_ and AUC_0–∞_, and a substantial 17.5-hour increase in t_1/2_ and decrease in apparent clearance of 2.5-fold.^20^ Finally, coadministration of bosutinib with multiple-dose ketoconazole (400 mg once daily) increased the C_max_, AUC_0–t_, and AUC_0–∞_ by 5.2-, 7.6-, and 8.6-fold, respectively; prolonged the t_1/2_ by 22.8 hours; and decreased apparent clearance by 9-fold.^17^

In conclusion, CYP3A4 inhibition by concomitant administration of ketoconazole with ponatinib increased ponatinib plasma AUC_0–∞_ and C_max_, but did not affect t_max_ or t_1/2_. Importantly, exposure to the CYP3A4-mediated metabolite AP24567 was marginal. Overall, these results suggest that caution should be exercised with the concurrent use of ponatinib and strong CYP3A4 inhibitors and that a ponatinib dose decrease to 30 mg daily, from the 45 mg daily starting dose, could be considered.
